# Mid-Term Outlook Following Modified Senning's Operation for the Correction of Transposition of the Great Arteries: A Case Series and Review of Literature

**DOI:** 10.7759/cureus.36770

**Published:** 2023-03-27

**Authors:** Vishal V Bhende, Tanishq S Sharma, Krishnan Ganapathy Subramaniam, Ashwin S Sharma, Amit Kumar, Purvi R Patel, Gurpreet Panesar, Kunal A Soni, Kartik B Dhami, Nirja P Patel, Hardil P Majmudar, Sohilkhan R Pathan

**Affiliations:** 1 Pediatric Cardiac Surgery, Bhanubhai and Madhuben Patel Cardiac Centre, Bhaikaka University, Karamsad, IND; 2 Community Medicine, SAL Institute of Medical Sciences, Ahmedabad, IND; 3 Pediatric Cardiac Surgery, Sri Padmavati Pediatric Heart Centre, Sri Venkateswara Institute of Medical Sciences (SVIMS) Campus, Tirupati, IND; 4 Internal Medicine, Gujarat Cancer Society Medical College, Hospital and Research Centre, Ahmedabad, IND; 5 Pediatric Cardiac Intensive Care, Bhanubhai and Madhuben Patel Cardiac Centre, Bhaikaka University, Karamsad, IND; 6 Pediatrics, Pramukhswami Medical College & Shree Krishna Hospital, Bhaikaka University, Karamsad, IND; 7 Cardiac Anaesthesiology, Bhanubhai and Madhuben Patel Cardiac Centre, Bhaikaka University, Karamsad, IND; 8 Clinical Research Services, Bhanubhai and Madhuben Patel Cardiac Centre, Bhaikaka University, Karamsad, IND

**Keywords:** myocardial protection, cardiac surgical procedure, survival analysis, transposition, chd: congenital heart disease

## Abstract

At the time of writing, two patients who underwent modified Senning’s operation (MSO) for the treatment of transposition of great arteries (TGAs) were followed up. At the time of surgery, the patients were three months and 15 years old, respectively. The duration of the follow-up was three years, during which there was a good prognosis, and hence no further invasive treatments were required. There was normal functioning of the right ventricle (RV) in both patients, with the exception of a minor baffle leak in the three-month-old patient. At the annual three-year follow-up, the tricuspid regurgitation (systemic atrioventricular valve) status was moderate in the three-year-old child and mild in the 18-year-old girl. Both patients maintained sinus rhythm and are assigned classification as New York Heart Association (NYHA) Classes I and II.

This study aims to assess the midterm outlook after MSO in order to identify and manage future long-term complications. Our report shows a positive outcome in terms of survival and functional activities among children with d-TGA; however, there is a strong need for future research to evaluate the prognosis in the long term (LT) and to assess the functioning of RV.

## Introduction

Transposition of great arteries (TGA) is a congenital heart defect (CHD) in which there is no connectivity between the great arteries (aorta and pulmonary artery) and their anatomically normal ventricles [1]. Ake Senning pioneered the switch operation (SO) performed in the atrium, as an invasive solution to TGA, in 1958 [2]. This treatment significantly improved the prognosis of TGA patients. An atrial baffle from the patient's atrial septum (AS) was used in the modified Senning’s operation (MSO) to direct the deoxygenated blood to the opposite atrioventricular valve and ventricle. Mustard described the alternative for venous return direction in 1963 [3], including AS removal and an artificial or pericardial baffle. About 90% mortality has been recorded among untreated TGA patients, particularly in the first year following birth [4]. Many TGA patients have reached adulthood thanks to the atrial switch operation (SO). However, since the mid-1980s, fear of future complications has led to the arterial switch operation (ASO) replacing the SO [5]. Nonetheless, given the large number of long-term (LT) follow up of Senning's procedure (SNP) survivors, their complications up to and after adulthood have gained more research interest [5,6]. This article also determines the effects of having the morphological right ventricle (RV) act as a systemic ventricle [1]. The main reason for such high mortality is the delayed diagnosis of CHD among developing countries, which results in patients presenting for surgery after the ideal period in which complete anatomical correction is possible.

Among d-TGA patients, the aortic semilunar valve is commonly seen right to the pulmonic valve or the pulmonary valve, among discordant ventriculoarterial and concordant atrio-ventricular connections [7,8]. The left ventricle (LV) of d-TGA patients becomes ineligible for ASO, even though the patient lives beyond five years, limiting the options for surgery to ASO with mechanical LV assist and ASO with LV retraining [7]. Though there is a good functional outcome and enhanced quality of life (QOL) for TGA patients treated with SO during childhood and adulthood [9], sudden death due to the risks of using the RV as the systemic ventricle does exist, along with RV dilatation, RV dysfunction, arrhythmias, sinus node dysfunction (SND), baffle leaks (BFLs), and pulmonary pathway and/or systemic pathway obstruction [10]. This article assesses the midterm outlook following the MSO in children with d-TGA. It also seeks to identify the factors contributing to post-surgical complications and evaluates the functional activities of children with d-TGA after surgery.

## Case presentation

The Institutional Ethics Committee (IEC-2) of HM Patel Center for Medical Care and Education, Anand, Gujarat, India, clarified this case study of any ethical issues (Approval No. IEC/BU/2022/Cr. 68/314/2022).

**Figure 1:**
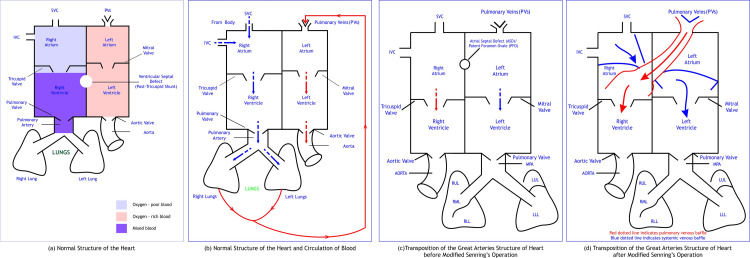
Schematic representation of the normal structure of heart and TGA heart before and after modified Senning's operation. TGA: transposition of the great arteries, SVC: superior vena cava. Image Credits: Dr. Vishal V. Bhende.

The legal guardians of the children gave informed consent following which a thorough history was taken, medical records and follow-up data were reviewed, and a thorough clinical examination was conducted. New York Heart Association (NYHA) norms were used for the classification of patients. A 12-lead electrocardiogram (ECG) was performed to detect cardiac arrhythmias. To begin this discussion, a diagram depicting the normal and the transposition of great arteries pre- and post-modified Senning's operation is shown in Figure 1. The patients' details are described in Table 1.

**Table 1:** Description of the cases. CPB: cardiopulmonary bypass, ACC: aortic cross-clamp.

S. no	Parameters	Case 1	Case 2
01	Age	Three months	15 years
02	Sex	Male	Female
03	Weight	3 kg	35 kg
04	Height	50 cm	159 cm
05	Diagnosis	d-TGA, moderate-sized ostium secundum atrial septal defect, shunting left to right, moderate-sized patent ductus arteriosus, intact inter-ventricular septum, left ventricle is regressed.	d-TGA large ostium secundum atrial septal defect, right to left shunt, left ventricle mass is regressed.
06	Surgery	Modified Senning’s operation + patent ductus arteriosus (PDA) ligation	Modified Senning's operation
07	Ventilator hours	13 days,9 h 30 min	72 h
08	Length of ICU stay	Eight days	Seven days
09	Hospital stay	28 Days	12 Days
10	CPB time	214 min	220 min
11	ACC time	181 min	153 min
12	2D echocardiography (echo) at discharge	Unobstructed flow in the pulmonary venous and systemic venous baffle. Small residual shunt between baffles, shunting from the systemic venous baffle to the pulmonary venous baffle. Mild to moderate tricuspid valve regurgitation. The right ventricle function is good. Inter-atrial obstruction was noted in the pulmonary venous baffle, gradient of 20/15 mmHg (peak/mean).	Unobstructed flow in pulmonary and systemic venous baffles. Mild right ventricle dysfunction. Mild pulmonary stenosis with a peak gradient of 24 mmHg.

Case 1

The first case involved a three-month-old male child weighing 3 kg with a height of 50 cm who presented with progressively worsening cyanosis and dyspnea on exertion. TGA, small atrial septal defect (ASD), and intact inter-ventricular septum (IVS) were all confirmed by clinical investigations, chest x-ray, and echo. The oxygen saturation ranged from 51% to 57%, and the hematocrit was 66%.

Case 2

The second case involved a 15-year-old female adolescent girl weighing 35 kg with a height of 159 cm who presented to the cardiac outpatient clinic with a history of dyspnea on exertion since one month of age and cyanosis during exertion since five years of age. She had CHD before the age of one month.

Operative procedure

The aim of the atrial switch, also known as the SNP, is to create an unobstructed pathway from the superior and inferior vena cava to the pulmonary artery via the mitral valve and left ventricle, and from the pulmonary veins to the aorta via the tricuspid valve and right ventricle. The principles used in the pathway’s construction are to use as much native tissue as possible, to avoid conduction pathways, and to use in-situ tissue to allow for growth. The initial steps involve opening the pericardium slightly to the left of the midline to make more pericardium available on the right side for the creation of a locally placed baffle, cannulating the superior vena cava (SVC) and inferior vena cava (IVC), retaining the pericardial reflection around the pulmonary veins, and separating the phrenic nerve from the pericardium to avoid injury during the pulmonary baffle creation. The inter-atrial groove is also dissected to make more of the left atrium (LA) available for opening up the pulmonary pathway and walling off the pulmonary veins from the mitral valve.

Following the opening of the right atrium (RA) behind the atrioventricular groove parallel to the vena cava line of orientation, stays are placed to the anterior and posterior of the right atrial flap. The atrial septum is first examined; if there is a large ASD, as most patients undergoing a Senning procedure are likely to have, a patch of polytetrafluoroethylene (PTFE) or pericardium may be required to augment the tissue.

*First Layer* 

The first layer separates the pulmonary veins from the mitral valve and forms the floor of the systemic baffle or the roof of the pulmonary baffle. If the atrial septum is small, a cut back into the superior limbus may be necessary to avoid gradients at the superior vena cava to the mitral valve pathway. This may occasionally entail going outside the heart, which must be inspected and closed from outside the right atrium (RA). Suturing begins from the base of the left atrial appendage anterior to the left pulmonary veins and proceeds either side superiorly across the LA roof or inferiorly posterior to the mitral annulus to reach the posterior atrial septal tissue.

Second Layer

This layer sutures the posterior right atrial flap to the anterior atrial septal tissue, baffling the superior and inferior vena cava blood; if necessary, a patch may be added to this layer. Opening up the coronary sinus and suturing inside the mouth of the coronary sinus anteriorly help avoid the bundle and widen the inferior vena cava pathway. This suturing begins at either end and ends in the middle of the anterior atrial septum. Squeezing the snuggers and briefly clamping the SVC and IVC venous lines help in distending the pathway to assess adequacy and identify any potential leak sites. The systemic pathway is now complete.

Third Layer

**Figure 2:**
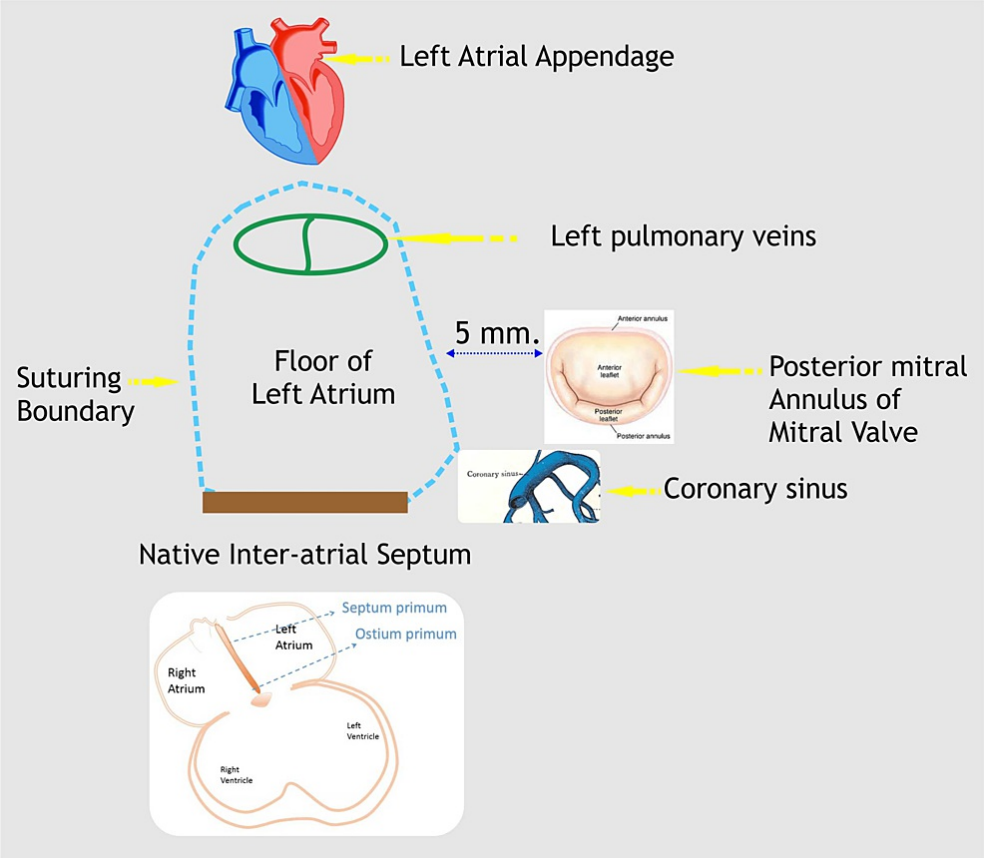
Suturing perimeter of the first step of modified Senning’s operation. Image Credits: Dr. Vishal V. Bhende.

To keep the pulmonary veins open, a few tacking sutures can be placed between the pulmonary veins and the pericardium. The suturing begins from the superior aspect of the left atrial incision and transitions onto the in-situ pericardium and climbs across the superior vena cava, carefully avoiding the sino-atrial (SA) node, and then continues on to the anterior aspect of the right atrium toward the middle; the inferior aspect of the pulmonary venous opening is likewise sutured to the pericardium, which goes across the inferior vena cava to the right atrial flap meeting the superior suture. A sucker in the right atrial appendage helps in return control. The blood is effectively baffled after this layer is completed. Extending the chamber by clamping the right atrial appendage vent allows leaks from the pulmonary venous baffle to be identified. Atrial and ventricular pacing wires are placed, the right pleura is drained, and the patient is gradually weaned off CPB (Figure 2) (Videos 1-3).

**Video 1:** World's first modified Senning's operation 3D medical animation. Video Credits: Dr. Vishal V. Bhende.

**Video 2:** Modified Senning's operation tips and tricks. Video Credits: Dr. K. S. Ganapathy.

**Video 3:** Modified Senning's operation video abstract. Video Credits: Dr. Vishal V. Bhende.

**Figure 3:**
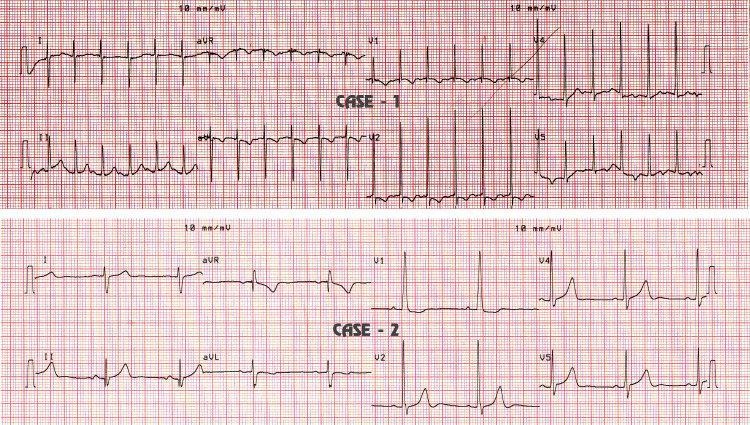
Electrocardiographic strips for cases 1 and 2 at mid-term follow-up for modified Senning’s operation depicting normal sinus rhythm (NSR).

There were no post-operative deaths and the need for re-operation or re-intervention. Patients were subjected to 12-lead ECGs during the follow-up period. During the three-year follow-up period, both patients had normal sinus rhythm (Figure 3).

**Figure 4:**
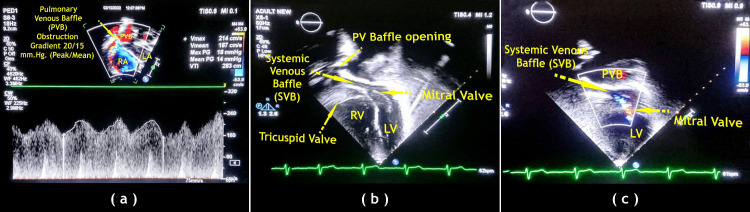
Post-operative 2D echocardiography images of modified Senning’s operation. (a) Case 1. (b) and (c) Case 2. Image Credits: Dr. Vishal V. Bhende.

**Figure 5:**
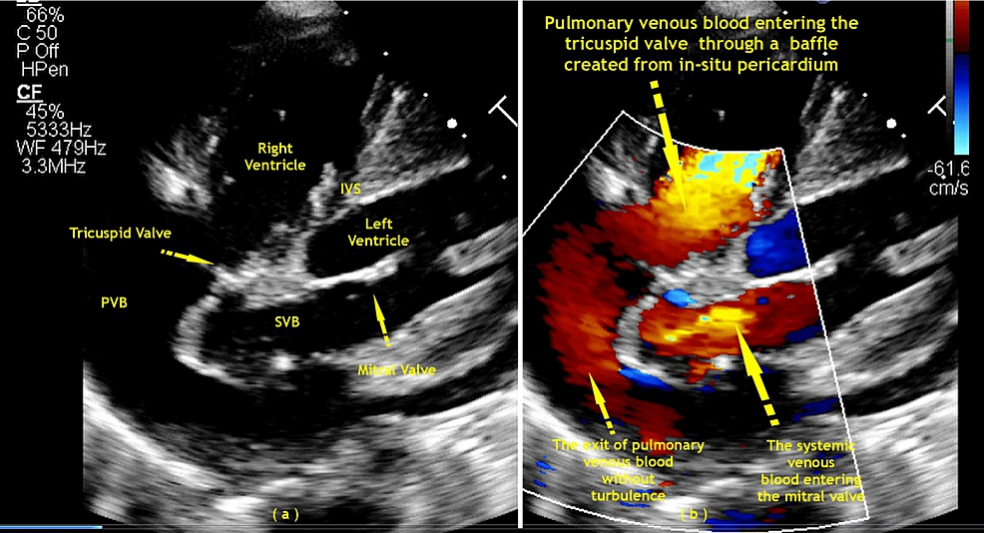
Post-operative 2D echocardiography apical four-chamber images with the posterior tilt of modified Senning’s operation in case 2. (a) 2D echo and (b) color Doppler. PVB: pulmonary venous baffle, SVB: systemic venous baffle, IVS: inter-ventricular septum. Image Credits: Dr. K. S. Ganapathy.

A follow-up 2D Doppler echo was performed every year. In case 1, pulmonary venous baffle obstruction was discovered. The gradient was 30/22 mmHg (peak/mean) at the first-year follow-up and decreased to 20/15 mmHg (peak/mean) at the third-year follow-up. The tricuspid valve regurgitation (systemic atrioventricular valve) was moderate, and the right ventricle function was good. In case 2, moderate right ventricular dysfunction was present with mild tricuspid valve regurgitation and minimal pulmonary valve stenosis, with a peak gradient of 16 mmHg noted with mild pulmonary valve regurgitation (Figures 4, 5).

## Discussion

The SNP was the first surgical solution for TGA. The SNP increased the length of survival in patients with TGA. The SNP uses an atrial switch to divert venous drainage, which was later substituted by the arterial switch operation, which provided anatomic and physiologic correction of the problem. Even though there are generally positive results from this correction, several complications do exist [11].

The midterm results of our patients were satisfactory. There were no fatalities among our patients, and no re-operations or re-interventions were required. Likewise, Talwar et al. [7] reported nil early or late mortality in patients with TGA, and no one required re-operations during the follow-up period. On the other hand, Maluf [9] recorded single death (2.5%) in his long-term follow-up. Dos et al. [5] registered a 5.1% of late mortality rate after SO, with 42.8% of sudden death much similar to the other studies [12]. At three years, both of our patients survived, like the study done by Talwar et al. [7] and Maluf [9], who registered nil mortality at five, ten, and fifteen years after intervention. Dos et al. [5], later, in a similar study, documented 99% and 95% of survival at ten and fifteen years, respectively. In a study of 20 years follow-up, Lange et al. [13] registered a 91% survival rate following the intervention.

Following Senning's operation, arrhythmias are a common complication. Byrum et al. [14] proved that re-entry mechanisms associated with multiple suture lines in the atrium are the primary causative factor for symptomatic arrhythmia, and the SND is a result of direct injury to the node or its arterial blood vessel. In our article, it can be seen that both patients' sinus rhythms were preserved.

The deterioration of systemic RV activity following a Senning's operation is a primary issue [9]. LT survival is primarily found by the performance of the tricuspid valve and RV against the systemic afterload and blood pressure. Out of the two cases, only case 2 had mild right ventricular dysfunction. Sarkar et al. [15] and Wells and Blackstone [6] found RV dysfunction in 1% of their subjects and documented findings on the same line. But Dos et al. [5] documented an increased occurrence of RV dysfunction (14.6%) in their subjects with TGA, and Kirjavainen et al. [16] reported an even higher incidence (28%) in their research of LT outcomes after a MSO. The difference in our results compared to the literature may be due to the age of our d-TGA patients, which was much younger compared to others, and the short duration of follow-up as RV dysfunction happens in aged as a late complication. Alternatively, Dos et al. [5] demonstrated that 2D echo is an inferior diagnostic method compared to magnetic resonance imaging (MRI) and 3D echo in diagnosing RV function as they provide more intricate information. At the three-year follow-up point, case 1 had moderate tricuspid regurgitation, and case 2 had mild tricuspid valve regurgitation. Maluf [9], Szymański et al. [17], and Kammeraad et al. [18] found marked tricuspid regurgitation in their studies with 22.8%, 33.3%, and 20%, respectively, following Senning's operation.

Reoperations following the Senning procedure are frequently caused by baffle-related complications (BRCs) [9]. Hörer et al. [19] documented similar complications in 5.4% of their patients following 18.2 years of surgery following venous baffle leaks or stenosis. Similarly, Szymański et al. and Maluf registered BRC among 3.3% and 5.4% of their patients, respectively, following a MSO. A small residual shunt between the systemic and the pulmonary venous baffles was observed in our case 1, but it was insignificant and did not necessitate reoperation or re-intervention. On room oxygen, both patients in our study maintained a systemic oxygen saturation (SOS) of ≥95%. Both patients had NYHA class I. Helbing et al. [20], Wells and Blackstone [6] documented 95%, and Maluf [9] documented 100% of their TGA patients with NYHA classes I and II during LT follow-up, much similar to our study. Furthermore, Talwar et al. [7] documented that all their SNP group was in NYHA class I post-operatively, had SOS of more than 95% on room air, and all cardiac medications were stopped. In this report, 50% of the patients (case 1) continued their cardiac medications and diuretics, for the duration of their follow-up. Dos et al. [5] found a similar result, as their TGA patients received postoperative medical treatment for the duration of their follow-up, including digitalis (8.8%), diuretics (4.4%), angiotensin-converting enzyme (ACE) inhibitors (6.6%), and anti-arrhythmic drugs (4.4%).

The major milestones in the management of TGA are described in Table 2.

**Table 2:** Milestones in transposition of the great arteries (TGAs).

Year	Author	Contribution
1797	Baillie and Wardrop [21]	First morphological description of TGA
1814	Farre [22]	First used the term TGA
1948	Hanlon and Blalock [23]	Atrial septectomy-first palliative operation
1953	Lillehei and Varco [24]	Partial venous switch
1955	Baffes [25]	Baffes operation
1957	Senning [2]	Atrial switch using atrial flaps
1963	Mustard [3]	Atrial switch using pericardium
1966	Rashkind and Miller [26]	Introduced balloon atrial septostomy
1975	Jatene et al. [27]	Successful arterial switch
1976	Yacoub et al. [28]	Left ventricular training and two-stage arterial switch
1981	Lecompte et al. [29]	Lecompte maneuver
1984	Castaneda et al. [30]	Neonatal arterial switch
1989	Jonas et al. [31]	Rapid two-stage arterial switch

## Conclusions

We present the results for two pediatric patients who underwent a MSO for d-TGA. Patients with d-TGA were treated with a modified SNP and then evaluated three years later. The absence of mortality and improved functional status were satisfactory outcomes. However, regular follow-up is necessary to detect any arrhythmias and BRC. Further research is needed to assess long-term outcomes and right ventricular function in this subset of patients.
